# Cardiac lesions in sheep and goats with spontaneous *Clostridium perfringens* type D enterotoxemia

**DOI:** 10.1177/03009858261426544

**Published:** 2026-03-07

**Authors:** Emma H. Torii, Omar A. Gonzales-Viera, Jennine Ochoa, Todd Cornish, Nicolas Streitenberger, Roberto W. I. Olivares, Hannah Neer, Francisco A. Uzal, Asli Mete

**Affiliations:** 1 University of California, Davis, Davis, CA; 2 University of California, Davis, Tulare, CA; 3 University of California, Davis, San Bernardino, CA; 4 Mill Station Veterinary Service, Dixon, CA

**Keywords:** *Clostridium perfringens* type D, enterotoxemia, goats, heart, histology, histopathology, myocardial degeneration, myocardial edema, myocardial necrosis, sheep, small ruminants

## Abstract

A 12-year retrospective search identified 106 sheep and goats diagnosed with *Clostridium perfringens* type D enterotoxemia, and in which the heart was histologically examined. Twenty cases (20/106, 19%), including 10 sheep and 10 goats, had cardiac lesions that were presumed to be associated with enterotoxemia. The lesions included myocardial degeneration and/or necrosis (*n* = 16, 80%), hemorrhage (*n* = 17, 85%), and proteinaceous interstitial edema (*n* = 6, 30%). Myocardial degeneration and/or necrosis was more frequent in goats (10/10, 100%) compared with sheep (6/10, 60%). Hemorrhage was more frequent in sheep (10/10, 100%) compared with goats (7/10, 70%). Myocardial proteinaceous interstitial edema was exclusive to sheep. Cardiac lesions occur in spontaneous cases of *C. perfringens* type D enterotoxemia in small ruminants and may play a role in the clinical signs and/or the demise of the animal.

*Clostridium perfringens* is an anaerobic, spore-forming, gram-positive bacterium that is classified into 7 toxinotypes (A-G) based on the production of 6 major toxins: alpha (CPA), beta (CPB), epsilon (ETX), iota, enterotoxin, and necrotic enteritis B-like toxin.^22^ *C. perfringens* type D encodes CPA and ETX and is one of the most common causes of enterotoxemia in ruminants.^12,14,16,19^ A major predisposing factor is abrupt dietary change, especially to a diet rich in rapidly fermentable carbohydrates, which promotes bacterial proliferation and toxin production.^10,12,16,19,26,29^ Outbreaks have also occurred in extensive grazing systems without recent dietary changes. In these cases, other predisposing factors such as a heavy endoparasite burden (coccidia, tapeworms, or lungworms), cold environmental stress, and netobimin overdose may also contribute.^25,26,29,32^

ETX is the major virulence factor for *C. perfringens* type D, although other toxins have been suggested to potentially play a role in the disease development.^14,18,19,25,34^ ETX is part of the aerolysin family of pore-forming toxins.^12,19,25,34^ This toxin is secreted as a relatively inactive pro-toxin and is cleaved into its active form via enzymatic activity by trypsin or other proteases.^12,14,16,18^ The activated toxin is absorbed and distributed systemically through the blood to target organs, and binds to a receptor on endothelial cells and other cell types, with resultant transmembrane pore formation and cell necrosis.^12,16,18,19,25,34^ The specific target receptor is yet to be definitively identified, but recently, the myelin and lymphocyte (MAL) protein has been proposed to be the target receptor.^16,18,24^

The clinical signs and lesions of enterotoxemia have been characterized in sheep, where it is considered a systemic disease with minimal or inconsistent intestinal involvement.^12,14,25,26^ Clinical signs include increased respiratory efforts, recumbency, lethargy, incoordination, paddling, bleating, convulsions, blindness, opisthotonos, and less commonly, diarrhea. Common postmortem gross lesions include hydropericardium with or without fibrin, pulmonary edema, serosal hemorrhages, and less frequently, cerebellar coning and focal symmetrical encephalomalacia. Microscopic lesions include myocardial hemorrhage in the heart, proteinaceous edema in the lungs and myocardium, and proteinaceous perivascular and intramural edema in vessels in the brain and lungs, indicative of increased vascular permeability.^5,14,16,26,28,31,33,34^ Cardiac lesions have been reported in sheep experimentally inoculated with *C. perfringens* type D,^14,16^ but there is limited information on cardiac lesions in natural cases of the disease in this species. The role ETX plays in the pathogenesis of cardiac lesions in sheep are poorly understood, but has been suggested to be secondary to effects of ETX on endothelial cells.^16^

Clinical signs and lesions of enterotoxemia are less well characterized in goats.^25,29,33^ In contrast to sheep, enteric involvement is common,^14,25,30^ and the disease can present as an enterotoxemic form, enteric form, or a combination of the two.^12,14^ Commonly reported gross and histological lesions in goats include fibrinohemorrhagic to fibrinonecrotizing enterocolitis, hydropericardium with or without fibrin clots, pulmonary edema, and subendocardial and epicardial hemorrhages.^4,12,14,19,25,26,29,30,32,34^ Perivascular and intramural vascular proteinaceous edema is less common in the brains of goats compared with sheep.^19^

Although there are a couple of experimental studies describing cardiac lesions in sheep with experimental enterotoxemia in detail,^14,16^ no significant lesions in the heart tissues of the experimental goats have been described.^14,28^ Possible cardiac lesions have been rarely described in spontaneous cases of type D enterotoxemia in small ruminants.^17,21,35^ Here, we describe gross and histological cardiac lesions in sheep and goats with spontaneous type D enterotoxemia.

## Materials and Methods

### Data Collection and Analysis

A retrospective search of the California Animal Health and Food Safety Laboratory System (Davis, Tulare, and San Bernardino branches) archives between January 1, 2013, and December 13, 2024, was conducted to identify sheep and goats with a diagnosis of *C. perfringens* type D enterotoxemia. Selection criteria were positive ETX and negative CPB detection by ELISA in the small and/or large intestinal contents and cases in which the heart was histologically examined. Thereon, the selected case reports were analyzed and the signalment, clinical history, ancillary tests to aid in the diagnosis, and gross and microscopic lesions, with particular attention to those in the heart, were recorded. The hematoxylin and eosin-stained heart tissue sections of these cases where the examining pathologist mentioned any cardiac lesions were re-examined by 2 board-certified anatomic pathologists (EHT and AM). The slides were assessed for degeneration and/or necrosis, inflammation, eosinophilic (proteinaceous) interstitial edema, and hemorrhage, and the location was noted (ie, myocardial, endocardial, and/or epicardial). These lesions were subjectively evaluated for severity (minimal/mild, moderate, marked, depending on the estimated amount of tissue affected and number of foci), distribution (focal, multifocal, and diffuse), and chronicity. The presence of myocardial degeneration and/or necrosis was identified if cardiomyocytes had one or more of the following findings: hypereosinophilic sarcoplasm, loss of cross-striation, fragmented sarcoplasm, contraction band necrosis, and/or pyknosis or loss of nuclei. The degeneration and/or necrosis was considered minimal/mild if they were rare to few foci and involved individual or small clusters of cardiomyocytes; moderate if it involved few, small to medium foci; and marked if there were regional, larger and/or numerous foci. The inflammation was categorized as absent, neutrophilic, lymphocytic, histiocytic, or a combination thereof. Necrosis was considered acute if there was absence of inflammatory cells or predominantly neutrophilic inflammation; subacute if there was lymphocytic and/or histiocytic inflammation, or a combination of neutrophilic and lymphohistiocytic inflammation; and chronic if there was fibrosis. Cases were identified as having proteinaceous interstitial edema if there was eosinophilic, homogeneous, fluid surrounding blood vessels. The edema was considered minimal/mild when there were multiple, rare foci of interstitium involved; moderate if it involved more numerous, small to medium, regional foci; and marked if there was diffuse involvement. The hemorrhage was classified as minimal/mild if it involved small and/or few foci with a few extravasated erythrocytes mildly expanding the interstitium; moderate if it involved a few, medium-sized foci; and marked if there were numerous and large foci expanding the interstitium and separating individual cardiomyocytes. Cases in which there were confounding factors that could have caused the death, such as clinical history of cardiotoxin exposure or systemic infection, such as sepsis and/or bacteremia, were excluded. Cases that had *Sarcocystis* sp. burdens associated with inflammatory changes were noted. A Pearson’s χ^2^ test was performed to evaluate the statistical significance between the lesions in the goat and sheep. The results were considered to be statistically significant if *P* < .05.

### Epsilon Toxin Detection

A capture ELISA for *C. perfringens* CPB and ETX was performed on small and/or large intestinal contents using a commercial kit (Bio-X), following the manufacturer’s instructions. This kit detects concentrations of ETX associated with disease, but not small amounts of the toxin that are below the threshold to produce disease. CPA results were not included in the ELISA testing of all cases. Since positive ETX, with exclusion of CPB, is the confirmatory diagnosis for type D enterotoxemia, the CPA results in this subpopulation are not included here.

### Other Ancillary Tests

Ancillary tests were done according to the California Animal Health and Food Safety Laboratory System standard operating procedures at the discretion of the pathologist performing the postmortem examination of submitted animals and/or tissues at the time, and varied between each case. Most commonly, these included liver heavy metal analysis (lead, manganese, iron, mercury, arsenic, molybdenum, zinc, copper, and cadmium) and selenium analysis via inductively coupled plasma—mass spectrometry, aerobic culture of lung and/or liver, fecal float or modified McMaster’s fecal egg count, *Salmonella* spp. PCR on intestinal contents, and anaerobic culture of the intestinal contents. Selenium values were classified as low when it was <0.25 ppm and were considered marginally low at 0.1–0.24 ppm and deficient at <0.1 ppm; these values were adapted from previously published data.^20^ If any tests to exclude myocardial toxins were performed (ie, ionophore screen via high-spectrum liquid chromatography or oleander glycoside testing via liquid chromatography-tandem mass spectrometry), this was also included in the results of this study.

## Results

### Data Analysis and Case Selection

The California Animal Health and Food Safety Laboratory System database search retrieved 109 necropsy submissions of sheep and goats with a diagnosis of type D enterotoxemia. Heart tissue was histologically examined in 106 of these submissions (32 sheep and 74 goats). Of these, 18 sheep and 17 goat cases had heart lesions, and the hematoxylin and eosin-stained slides of the heart of these 35 cases were re-examined and evaluated for this study. Ten cases were excluded due to concurrent findings that could have contributed to the cardiac lesions. Five additional animals had lesions that were too subtle for accurate characterization and were also excluded. Overall, 20 cases (10 sheep and 10 goats) were included in the study and consisted of 19 necropsies performed by the examining pathologist at California Animal Health and Food Safety Laboratory System, and 1 collection of tissues collected during a field necropsy by the submitting veterinarian.

All of the 20 cases included had clinical histories compatible with enterotoxemia, including sudden death, neurological clinical signs, and/or diarrhea (goat only). All but one case had gross and/or histopathologic findings compatible with ETX enterotoxemia, including hydropericardium, cerebellar coning, vasculocentric edema in the brain, pulmonary edema, enteritis, and/or colitis. None of the cases had a history of intracardiac euthanasia or cardiopulmonary resuscitation efforts.

### Signalment and Clinical Findings

The 20 cases consisted of 10 sheep and 10 goats. Ten were juvenile (<1-year-old) and 10 were adults (≥1-year-old), with ages ranging from 3 weeks to 5 years old (although the exact age was not mentioned for 2 animals). Eleven were female, 6 were male, and the sex was not mentioned for 3 animals. The signalments and duration of clinical signs are summarized in Table 1, and in-depth data are provided in Supplemental Table S1. The duration of illness varied from 0 to 3 days, and it was not mentioned for one case. The most common clinical presentation was diarrhea in goats (7/10), and death without premonitory signs in sheep (6/10). Other reported clinical signs included recumbency, thrashing, weakness, anorexia/inappetence, lethargy, mild fever, hypothermia, and incoordination. Two goats were euthanized following a history of diarrhea and anorexia, and all other animals died spontaneously.

**Table 1.** Signalment and duration of clinical signs of sheep and goats that died of type D enterotoxemia.

Characteristic	Sheep No. (%)	Goat No. (%)	Total No. (%)
Total	10/20 (50%)	10/20 (50%)	20/20 (100%)
Age			
Juvenile	9/10 (90%)	1/10 (10%)	10/20 (50%)
Adult	1/10 (10%)	9/10 (90%)	10/20 (50%)
Sex			
Female	2/10 (20%)	9/10 (90%)	11/20 (55%)
Male	5/10 (50%)	1/10 (10%)	6/20 (30%)
Unknown	3/10 (30%)	0/10 (0%)	3/20 (15%)
Duration of illness			
0 days	6/10 (60%)	1/10 (10%)	7/20 (35%)
<1 day	2/10 (20%)	3/10 (30%)	5/20 (25%)
1 day	1/10 (10%)	3/10 (30%)	4/20 (20%)
2 days	0/10 (0%)	2/10 (20%)	2/20 (10%)
3 days	0/10 (%)	1/10 (10%)	1/20 (5%)
Unknown	1/10 (10%)	0/10 (0%)	1/20 (5%)

### Gross Findings

**Figure 1.**
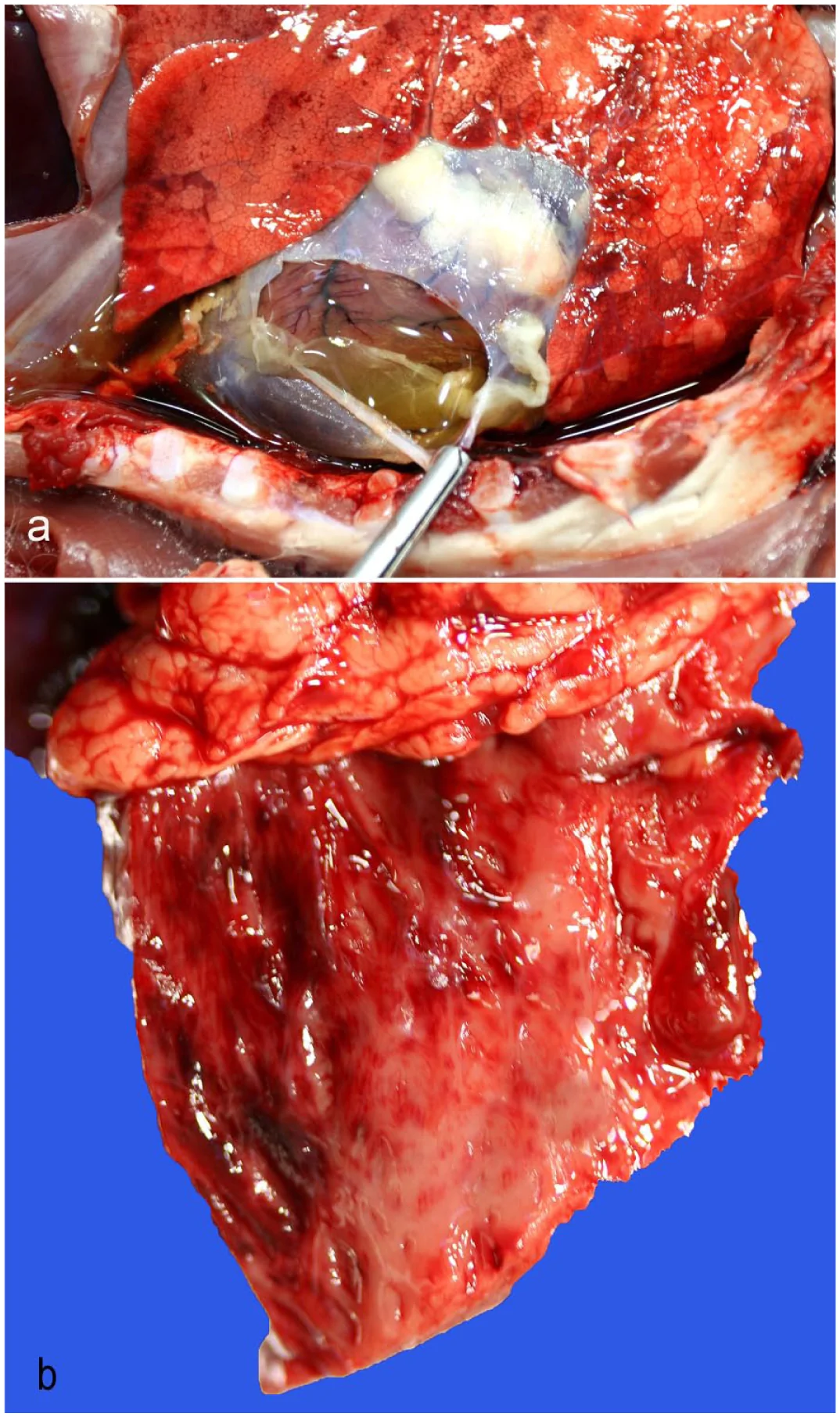
Macroscopic cardiac lesions of *Clostridium perfringens* type D enterotoxemia, lambs. (a) Pericardial sac expanded by a moderate amount of yellow, watery, and clear fluid with thick fibrin clots. (b) Multifocal endocardial hemorrhages.

A total of 7 sheep had gross cardiac lesions (Table 2), including hydropericardium with or without fibrin clots (*n* = 7) (Fig. 1a) and endocardial (*n* = 3) (Fig. 1b), epicardial (*n* = 2), and/or myocardial (*n* = 1) hemorrhages. One goat had a few petechiae in the papillary muscles. No other goats had gross cardiac lesions mentioned in the reports. Chi-square analysis indicated statistically significant difference in the presence of hydropericardium between the 2 species (*P* = .007).

**Table 2.** Gross and histological cardiac lesions associated with type D enterotoxemia in sheep and goats.

Lesions	Sheep No. (%)	Goat No. (%)	*P*-value*	Total No. (%)
Hydropericardium (gross ^a^)	7/10 (70%)	0/9 (0%)	.007	7/19 (37%)
Myocardial degeneration/necrosis (histologic)	6/10 (60%)	10/10 (100%)	.094	16/20 (80%)
Associated with inflammation	1/6 (17%)	6/10 (60%)	.2	7/16 (44%)
Edema (histologic)	6/10 (60%)	0/10 (0%)	.015	6/20 (30%)
Myocardial	6/10 (60%)	0/10 (0%)	.015	6/20 (30%)
Endocardial	0/10 (0%)	0/10 (0%)	^b^	0/20 (0%)
Epicardial	3/10 (30%)	0/10 (0%)	.2	3/20 (15%)
Hemorrhage (histologic)	10/10 (100%)	7/10 (70%)	.2	17/20 (85%)
Myocardial	8/10 (80%)	3/10 (30%)	.072	11/20 (55%)
Endocardial	7/10 (70%)	6/10 (60%)	>.9	13/20 (65%)
Epicardial	5/10 (50%)	0/10 (0%)	.039	5/20 (25%)

a Tissue only submission was excluded from the count.

b Statistical evaluation could not be performed as none of the animals had this lesion.

* *P* < .05 considered statistically significant.

### Histological Findings

**Figure 2.**
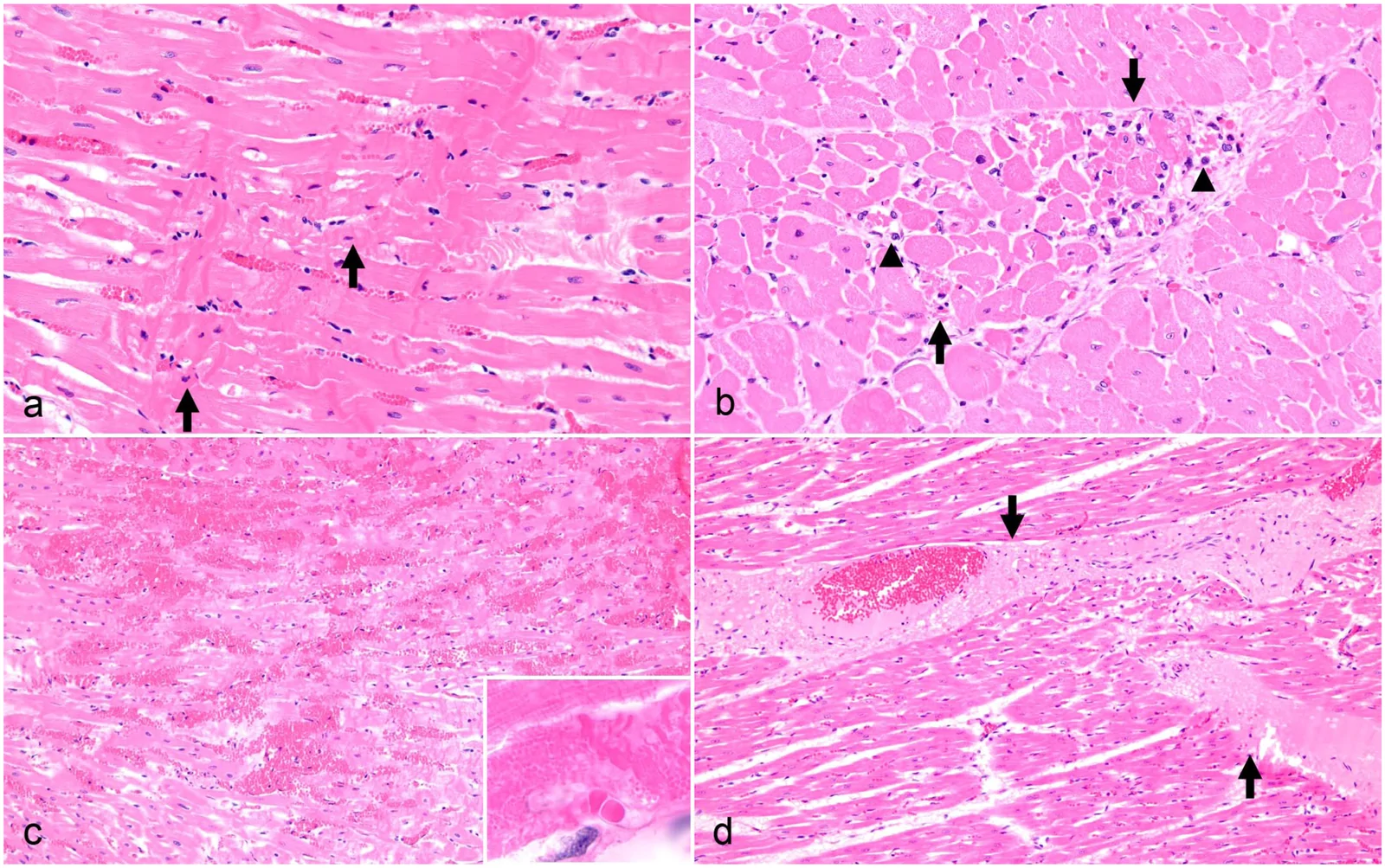
Heart microscopic lesions in goats and sheep with type D enterotoxemia, hematoxylin and eosin. (a) Goat with acute monophasic myocardial necrosis, longitudinal section. Affected cardiomyocytes show hypereosinophilia with fragmentation of the sarcoplasm, lack of cross-striations, pyknotic nuclei, and contraction band necrosis (arrows). The nuclei are pyknotic. (b) Goat with subacute myocardial necrosis, cross-section. There is a focus of cardiomyocytes with hypereosinophilia and fragmentation of the sarcoplasm (arrows), and mild infiltrates composed of histiocytes and/or hypertrophied, interstitial stromal cells (arrowheads). (c) Sheep with a locally extensive region of myocardial hemorrhage, longitudinal section. There is contraction band necrosis in the adjacent cardiomyocytes. Inset: High magnification of the contraction band necrosis. (d) Sheep with expansion of the interstitium by smooth eosinophilic (proteinaceous) edema (arrows), tangential section.

The number of heart sections available for examination from each animal varied from 2 to 9. Cardiac histological lesions are summarized in Table 2. A total of 16 animals (80%) had minimal/mild (*n* = 9) or moderate (*n* = 7) myocardial degeneration and/or coagulative necrosis (Fig. 2a–c), with a higher prevalence in goats (10/10, 100%) compared to sheep (6/10, 60%). In all animals with myocardial degeneration and/or necrosis, the lesions were acute (*n* = 12) to subacute (*n* = 4), randomly distributed, and monophasic. In 6 goats and 1 sheep, the necrotic foci were associated with mild inflammation. The inflammation in the goats with ≤1 day history of illness and one sheep with an unknown duration of illness was predominantly neutrophilic. In those where the duration of illness was >1 day, it was a predominantly histiocytic inflammation with fewer neutrophils and prominent interstitial stromal cells (Fig. 2b). In 5 sheep, the necrotic foci were often associated with hemorrhage (Fig. 2c), whereas in the other animals, hemorrhage and necrosis often occurred separately.

Proteinaceous interstitial cardiac edema (Fig. 2d) was observed in 6/10 sheep (60%), but in none of the goats. In 4 of these sheep, the edema was minimal/mild, and it was moderate in 2 sheep. The edema always involved the myocardial interstitium, and in 3 sheep, it was additionally observed in the epicardium. In 2 of these 6 sheep, there was also mild (*n* = 1) or moderate (*n* = 1) myocardial degeneration and/or necrosis, but not necessarily associated with regions of edema. All 6 sheep also had mild (*n* = 5) or marked (*n* = 1) myocardial (*n* = 4), endocardial (*n* = 3), and/or epicardial (*n* = 4) hemorrhage.

All sheep and 7 goats (*n* = 17) had hemorrhage in at least 1 site (myocardium, endocardium, or epicardium). Endocardial hemorrhage was most common (13/17, 76%), followed by myocardial (11/17, 65%) and epicardial (5/17, 29%) hemorrhage. The hemorrhage was mild in the majority of animals (*n* = 15), moderate in one sheep, and marked in another sheep. The hemorrhages were multifocal and random, except in 3 cases where they were in one small focus.

Chi-square analysis indicated statistical differences between sheep and goats with regard to cardiac proteinaceous edema (overall) (*P* = .015), myocardial proteinaceous edema (*P* = .015), and epicardial hemorrhage (*P* = .039). No statistical difference was observed with myocardial degeneration and/or necrosis (*P* = .094), epicardial edema (*P* = .2), hemorrhage (overall) (*P* = .21), myocardial hemorrhage (*P* = .072), or endocardial hemorrhage (*P* ≥ .9).

In all animals, other organs that were examined histologically by the pathologist at the time of the diagnostic workup included the intestines (small and/or large intestines), lung, liver, and kidney. The brain was examined in all sheep and 6 goats. Other examined tissues were the spleen (18), rumen (18), abomasum (16), skeletal muscle (14), lymph node (12), and adrenal gland (11). Nineteen of the 20 animals were reported to have histological lesions compatible with enterotoxemia in the brain, lungs, and/or intestines (Table 3 and Supplemental Table S1). Brain lesions were observed only in the sheep and included proteinaceous vasculocentric edema (6/10) and thalamic necrosis (1/10). Of the 6 sheep with perivascular edema of the brain, 4 also had myocardial edema. Other reported lesions included fibrinous, necrotizing, neutrophilic, and/or hemorrhagic enteritis and/or colitis (2/10 sheep and 9/10 goats), and pulmonary edema and/or congestion (6/10 sheep and 4/10 goats).

**Table 3.** Extracardiac histological lesions associated with type D enterotoxemia in sheep and goats.

Lesions	Sheep No. (%)	Goat No. (%)	Total No. (%)
Perivascular edema of brain	6/10 (60%)	0/10 (0%)	6/20 (30%)
Thalamic malacia of brain	1/10 (10%)	0/10 (0%)	1/20 (5%)
Fibrinous, necrotizing, and/or hemorrhagic enteritis and/or colitis	2/10 (20%)	9/10 (90%)	11/20 (55%)
Pulmonary edema and/or congestion	6/10 (60%)	4/10 (40%)	10/20 (50%)

### Ancillary Tests

Liver selenium determination was performed in all but one animal. Selenium was low in 7/19 animals, including 1 goat with deficiency (0.081 ppm), and 4 sheep and 2 goats with marginally low levels (0.14–0.24 ppm). Of these, 2 sheep and 2 goats with marginally low selenium had minimal/mild, acute (3) to subacute (1), monophasic myocardial degeneration/necrosis, and 1 goat with deficient levels had moderate, acute, monophasic myocardial degeneration/necrosis. The goat with deficient selenium had rare neutrophilic infiltration associated with foci of necrosis. The 2 other sheep with marginally low selenium levels had myocardial, interstitial proteinaceous edema and no myocardial degeneration and/or necrosis. There were no skeletal muscle lesions observed in the 5/7 animals with low selenium and for which skeletal muscle was histologically examined, except for one animal with mild, multifocal, lymphocytic myositis. In 9 other animals with mild (*n* = 4) to moderate (*n* = 5) myocardial degeneration and/or necrosis, the selenium level was adequate. Selenium analysis was not performed in the one other animal with myocardial degeneration and/or necrosis. In all animals, a heavy metal screen was performed on the liver. Copper and zinc deficiencies were found in 6/20 animals and 2/20 animals, respectively. Anaerobic culture on small and/or large intestinal contents were performed in 7/20 animals, and *C. perfringens* was cultured from 5/7 of these animals. An ionophore screen on the feed was performed in 1 animal, and oleander glycoside testing on the liver was performed in 2 animals. No ionophore or oleander glycoside were detected in the tested specimens. Signalment, clinical manifestations, cardiac and other lesions compatible with type D enterotoxemia, and other concurrent findings are presented in Supplemental Table S1.

## Discussion

Since *C. perfringens* can be a normal intestinal inhabitant, the detection of ETX in intestinal contents is necessary for the definitive diagnosis of enterotoxemia, along with consistent clinicopathological findings.^6,25,26,30,33^ In this study, all animals had ETX detected via ELISA in the small and/or large intestinal contents and consistent clinicopathological findings for enterotoxemia-associated death. Despite having minimal autolysis, one sheep that died without premonitory signs had no other lesions compatible with enterotoxemia. However, in some cases, sheep may not produce discernible gross or histological findings.^14,26,33^ In this sheep, enterotoxemia was the presumed cause of death based on the detection of ETX and the absence of other identified causes of death.

In prior published work, histologic cardiac lesions in suspected spontaneous cases of enterotoxemia have been rarely reported in goats, sheep, and camels albeit often without adequate testing for ETX.^2,17,21,35^ In addition, the provided histological images were of poor quality or did not match the description. We searched Google, PubMed, CAB Direct, Web of Sciences, and Scopus using the keywords “myocardial necrosis, cardiac necrosis, cardiopulmonary, cardiac, heart, enterotoxemia, and *Clostridium perfringens* type D” and found no further reported cases of histological cardiac lesions in small ruminants with spontaneous *C. perfringens* type D enterotoxemia. Excluding 3 experimental studies in sheep,^14–16^cardiac lesions in both experimental and spontaneous cases of enterotoxemia have otherwise been rarely documented in small ruminants, where the heart was often not examined histologically or was not mentioned to have been examined.^4–6,27,30–32^ In other experimental studies where the heart was histologically examined, no lesions were noted in goats.^14,28^ Another experimental study involving sheep described hydropericardium, but no histological cardiac lesions.^28^ Spontaneous cases in 2 goats showed no histological lesions in the heart.^1^

The experimental study that described histological cardiopulmonary lesions in detail in sheep found hydropericardium as the most frequent gross cardiac lesion.^16^ Histologically, there was cardiomyocyte degeneration and necrosis, and the most striking features were proteinaceous myocardial edema and occasional hemorrhage that often colocalized with necrosis.^16^ Another experimental study also described myocardial interstitial edema in sheep, but not in goats.^14^ In our study, hydropericardium and myocardial proteinaceous interstitial edema were only observed in sheep and not goats, and the result was statistically significant. It is well recognized that sheep and goats have different clinicopathological presentations in both experimental and spontaneous cases of *C. perfringens* type D, despite ETX being the major virulence factor in both species.^5,14,25,28,30,34^ Vasculocentric edema in the brain is more frequent in sheep^6,31,33,34^ and occurs at a reduced frequency in goats.^19^ Endothelial cells and other cell types within the central nervous system are the target of ETX, and cardiac and brain edema have been speculated to occur secondary to endothelial cell damage.^11,12,15,16^ In the current study, 4/6 of the sheep with proteinaceous myocardial interstitial edema also had perivascular proteinaceous edema in the brain, suggesting a similar pathogenesis. If the myocardial edema develops from a similar action of the ETX, then this may suggest that sheep are more susceptible to the effects of ETX on endothelial cells than goats. This has been previously hypothesized, given the difference in frequency of brain lesions between the 2 species.^27^ Another study suggested that the severity of myocardial edema may depend on the concentration of toxins entering the bloodstream.^15^ In addition, although both species can develop vasculocentric edema in the brain, it is often perivascular in sheep and more commonly intramural in goats.^19^ The reason for this is thought to be due to higher circulating levels of ETX in sheep.^19^ Hence, the difference in frequency of myocardial edema development between the 2 species could be due to differences in susceptibility and/or amount of ETX absorbed systemically.

Cardiomyocyte necrosis has been previously proposed to occur secondary to ischemia-hypoxia injury due to endothelial cell damage from ETX, given the frequent colocalization of the necrosis and edema.^16^ However, in our study, although some cases had hemorrhages colocalizing with the degeneration and/or necrosis, this was not always the case, especially in the goats. Furthermore, edema was not observed in any of the goats. Hence, the cardiomyocyte degeneration and/or necrosis could not be fully attributed to ischemia-hypoxia injury, as previously suggested. Direct toxic effects of ETX, which may be dose dependent, on cells other than endothelial cells, such as neurons and oligodendrocytes, have been suggested in previous studies.^10–12,14,18^ Thus, the possibility of ETX having a direct effect on cardiomyocytes resulting in necrosis cannot be completely excluded. To our knowledge, there has been no study looking at the direct effects of ETX on cardiomyocytes. Furthermore, it has previously been suggested that other toxins such as alpha and perfringolysin O may work synergistically with ETX and play a role in the pathogenesis of the disease.^8,19,25,28,30^ However, further studies are required to explore this hypothesis.

Seven of the animals in our study had inflammation associated with necrosis. This is different from a previous experimental study, in which only 1 of 12 sheep had mild neutrophilic inflammation.^16^ However, all experimental animals died or were euthanized within or at 24 hours after inoculation. In our study, some of the animals had clinical signs beyond 24 hours, and the duration of action of ETX is unknown in spontaneous cases. Furthermore, variations in the onset of clinical signs and gross and histological lesions have been noted in experimental studies in sheep, presumably due to differences in the duration of ETX’s action on tissues,^31^ and possibly due to breed/species susceptibility and levels of ETX production.

Differential diagnosis of the observed cardiac lesions, in particular the myocardial degeneration and/or necrosis, includes cardiotoxins such as certain plants (eg, oleander, avocado, yews, and white snakeroot) and ionophore toxicosis. Given the retrospective nature of the study, there was a lack of standardization of ancillary tests run on each case. In the majority of the cases, tests for potential cardiotoxins were not performed, and information on possible cardiotoxic plant exposure was not available. However, almost all the animals studied had clinical signs and pathological findings including extracardiac lesions compatible with enterotoxemia, which reduces the chances of a cardiotoxic agent causing the cardiac lesions. Given the inflammation, when observed, was thought to be secondary to the necrosis, other infectious etiologies were considered less likely.

One complicating factor of this study was the low liver selenium values in 7/19 animals; 5 of which (4 marginal and 1 deficient) had myocardial degeneration and/or necrosis. It is difficult to know the significance of these low selenium levels and their contribution to the observed myocardial lesions in the 5 animals. In nutritional myopathy associated with selenium deficiency with or without vitamin E deficiency, the cardiac necrosis is polyphasic, accompanied by macrophage infiltration, areas of fibrosis, and mineralization (which is often pronounced), and there is frequent skeletal muscle involvement.^3,7,9,23^ All the cases in this study had monophasic necrosis, none had mineralization, and none had skeletal muscle degeneration and/or necrosis. Thus, the animals in this study with low selenium levels did not have lesions compatible with nutritional myopathy. Furthermore, 10 animals with myocardial degeneration and/or necrosis (of which 5 were moderate severity) had liver selenium within the normal range. The reported selenium levels associated with nutritional myopathy varies between studies,^13^ and it is difficult to determine what level of selenium will develop nutritional myopathy.

There are several limitations to this study, given the retrospective nature as well as the heterogeneity of diagnostic cases. Firstly, there was a lack of standardization of the collection sites and the number of heart sections examined. In an experimental enterotoxemia study, the highest scores of necrosis and edema were observed in the AV nodes.^16^ Thus, it is possible that lesions were missed in some cases. Furthermore, the inclusion of cases depended on the mention of histological cardiac lesions in the original report, which may have created a selection bias. Although there were possible differences in the lesions observed in goats and sheep, statistically significant results were only noted with hydropericardium, cardiac proteinaceous edema (overall), myocardial proteinaceous edema, and epicardial hemorrhage. However, the overall number of animals included in this study was relatively small; hence, larger studies may be required to determine if statistically significant results are observed.

Here we report 20 sheep and goat fatalities from spontaneous type D enterotoxemia with associated cardiac lesions. Given the limited descriptions of cardiac lesions in experimental and spontaneous studies, myocardial lesions may be underdiagnosed in small ruminants with type D enterotoxemia. Under natural conditions, the disease may have a variable presentation. There may be differences in presentation between sheep and goats, although statistically significant results were only noted with hydropericardium, cardiac proteinaceous edema (overall), myocardial proteinaceous edema, and epicardial hemorrhage. Although the histological lesions described in this case series are relatively non-specific, we observed 20 out of 106 ETX-positive cases in which the histopathology of the heart was consistent with that reported in experimental cases.^16^ Hence, when myocardial degeneration and/or necrosis, hemorrhage and/or myocardial proteinaceous edema (especially in sheep) are observed, enterotoxemia should be considered a differential diagnosis.

## Supplemental Material

sj-pdf-1-vet-10.1177_03009858261426544 – Supplemental material for Cardiac lesions in sheep and goats with spontaneous Clostridium perfringens type D enterotoxemia Supplemental material, sj-pdf-1-vet-10.1177_03009858261426544 for Cardiac lesions in sheep and goats with spontaneous Clostridium perfringens type D enterotoxemia by Emma H. Torii, Omar A. Gonzales-Viera, Jennine Ochoa, Todd Cornish, Nicolas Streitenberger, Roberto W. I. Olivares, Hannah Neer, Francisco A. Uzal and Asli Mete in Veterinary Pathology
