# Supplementary material for: Cardiac lesions in sheep and goats with spontaneous Clostridium perfringens type D enterotoxemia
Source: Vet Pathol. 2026 Mar 7;63(5):871–8. doi: 10.1177/03009858261426544 (PMC13534770; doi:10.1177/03009858261426544)
Supplement: Supplementary file 1 [file sj-pdf-1-vet-10.1177_03009858261426544.pdf]

Supplemental Materials

Cardiac lesions in sheep and goats with spontaneous *Clostridium perfringens* type D enterotoxemia

Emma H Torii, Omar A Gonzales-Viera, Jennine Ochoa, Todd Cornish, Nicolas Streitenberger, Roberto WI Olivares,

Hannah Neer, Francisco A Uzal, Asli Mete

Supplemental table S1. Signalment, duration of illness, clinical signs, cardiac and other lesions compatible with type D enterotoxemia, and concurrent findings for each animal.

Case	Species	Age[a]	Sex	Duration of illness (days)	Clinical signs	Myocardial degeneration/ necrosis	Edema			Hemorrhage			Other lesions compatible with enterotoxemia	Other disease/condition(s)
							Myo	Endo	Epi	Myo	Endo	Epi		
**1**	Sheep	Juvenile	U	<1	Recumbent, then died soon after	Y – subtle, rare	N	N	N	Y	Y	Y	- Perivascular edema in the brain<br>- Pulmonary edema and congestion<br>- Hydropericardium with fibrin clots	- Marginally low liver selenium (0.22 ppm)<br>- Intestinal coccidiosis<br>- Abomasal nematodiasis with trichostrongyle eggs in feces
**2**	Sheep	Juvenile	U	0	None	N	Y	N	N	Y	N	Y	- Perivascular edema in the brain	- Marginally low liver selenium (0.14 ppm)<br>- Copper deficiency<br>- Coccidia oocysts in feces<br>- Trichostrongyle eggs in feces<br>- Cestodiasis<br>- Chronic bronchointerstitial pneumonia
**3**	Sheep	Juvenile	U	0	None	N	Y	N	N	Y	N	N	- None	- Marginally low liver selenium (0.14 ppm)<br>- Coccidia oocysts in feces<br>- Cestodiasis<br>- Liver granulomas
**4**	Sheep	Juvenile	M	0	None	Y – subtle and often associated with hemorrhage	Y	N	Y	N	Y	Y	- Perivascular edema in the brain<br>- Cerebellar coning<br>- Pulmonary edema<br>- Hydropericardium with fibrin	- Marginally low liver selenium (0.24 ppm)
**5**	Sheep	Juvenile	M	0	None	N	Y	N	Y	N	Y	N	- Perivascular edema in the brain<br>- Cerebellar coning<br>- Pulmonary edema<br>- Hydropericardium with fibrin clots	- None
**6**	Sheep	Juvenile	M	0	None	N	Y	N	Y	Y	N	Y	- Perivascular edema in the brain<br>- Pulmonary edema	- None
**7**	Sheep	Juvenile	M	<1	Thrashing, then died soon after	Y – often associated with hemorrhage	N	N	N	Y	Y	N	- Perivascular edema in the brain<br>- Fibrinonecrotizing enteritis<br>- Hydropericardium with fibrin	- Suboptimal liver zinc

**8**	Sheep	Juvenile	M	0	None	Y – subtle and often associated with hemorrhage	N	N	N	Y	Y	N	- Hydropericardium<br>- Neutrophilic enteritis<br>- Pulmonary edema	- Copper deficiency<br>- Zinc deficiency
**9**	Sheep	Juvenile	F	1	Fever, incoordination	Y – often associated with hemorrhage	N	N	N	Y	Y	N	- Thalamic malacia in the brain<br>- Hydropericardium with fibrin clot	- None
**10**	Sheep	Adult	F	U	U	Y – often associated with hemorrhage and/or with mild neutrophilic infiltration surrounding necrotic foci	Y	N	N	Y	Y	Y	- Pulmonary edema and congestion<br>- Hydropericardium with fibrin	- Copper deficiency<br>- Coccidia oocysts detected in feces<br>- Teladorsagiosis<br>- Marginally low ocular magnesium levels
**11**	Goat	Adult	F	0	None	Y	N	N	N	Y	N	N	- Necrotizing enterocolitis<br>- Pulmonary edema<br>- Hydrothorax with fibrin	- Copper deficiency<br>- Abomasal coccidiosis<br>- Trichostrongyle eggs detected in feces<br>- Trichuris eggs detected in feces
**12**	Goat	Adult	F	1	Diarrhea	Y – subtle, rare	N	N	N	N	Y	N	- Fibrinoneutrophilic enteritis<br>- Pulmonary edema and congestion	- None
**13**	Goat	Adult	F	1	Diarrhea, anorexia	Y- associated with mild lymphohistiocytic and neutrophilic infiltrates	N	N	N	N	N	N	- Necrotizing and mixed inflammatory enteritis<br>- Pulmonary edema	- Intestinal coccidiosis<br>- Lymphoplasmacytic abomasitis
**14**	Goat	Adult	M	1	Diarrhea, lethargy	Y - associated rare, small neutrophilic infiltrates surrounding necrotic foci	N	N	N	N	Y	N	- Fibrinonecrotizing enterocolitis	- Selenium deficiency (0.081 ppm)<br>- Copper deficiency<br>- Coccidia oocysts detected in feces
**15**	Goat	Adult	F	2	Diarrhea	Y – associated moderate histiocytic and fewer neutrophilic infiltrates surrounding necrotic foci	N	N	N	N	Y	N	- Necrotizing colitis	- Lymphoplasmacytic enterocolitis with mild crypt abscesses<br>- Coccidia oocysts detected in feces<br>- Mild neutrophilic rumenitis

**16**	Goat	Adult	F	<1	Found down, then died	Y – associated minimal neutrophilic infiltration into necrotic foci	N	N	N	N	N	N	-	Pulmonary edema	- Abomasal and intestinal coccidiosis<br>- Caseous lymphadenitis of one parotid lymph node<br>- Mild multifocal adrenal gland necrosis
**17**	Goat	Juvenile	F	<1	Hypothermic, then died soon after	Y – subtle, rare	N	N	N	Y	Y	N	-	Hemorrhagic enterocolitis	- Coccidia oocysts detected in feces
**18**	Goat	Adult	F	2	Diarrhea, lethargy, anorexia	Y – associated minimal, histiocytic infiltrates surrounding some of the necrotic foci	N	N	N	Y	Y	N	-	Necrotizing typhlocolitis and pleocellular enteritis	- Coccidia oocysts detected in feces<br>- Moderate pustular rumenitis<br>- Focally extensive suppurative bronchopneumonia
**19**	Goat	Adult	F	<1	Diarrhea	Y	N	N	N	N	N	N	-	Fibrinonecrotizing and neutrophilic enterocolitis	- Marginally low liver selenium (0.17 ppm)<br>- Copper deficiency<br>- Abomasal coccidiosis<br>- Trichostrongyle eggs detected in feces<br>- Neutrophilic rumenitis<br>- Mild lungworms
**20**	Goat	Adult	F	3	Intermittent diarrhea, inappetence	Y - associated mild to moderate histiocytic and rare neutrophilic infiltrates	N	N	N	N	Y	N	-	Fibrinonecrotizing and neutrophilic enterocolitis	- Marginally low liver selenium (0.14 ppm)<br>- Abomasal coccidiosis<br>- Trichostrongyle eggs detected in feces

Abbreviations: M, male; F, female; U, unknown; Y, yes; N, no; Myo, myocardium; Endo, endocardium; Epi, epicardium.

[a]Juvenile defined as <1-year-old and adult defined as ≥1-year-old
